# Ellagic acid inhibits the proliferation of human pancreatic carcinoma PANC-1 cells *in vitro* and *in vivo*

**DOI:** 10.18632/oncotarget.14811

**Published:** 2017-01-25

**Authors:** Hao Cheng, Chenglin Lu, Ribo Tang, Yiming Pan, Shanhua Bao, Yudong Qiu, Min Xie

**Affiliations:** ^1^ Department of General Surgery, Nanjing Drum Tower Hospital, The Affiliated Hospital of Nanjing University Medical School, Nanjing 210008, Jiangsu Province, China

**Keywords:** ellagic acid, pancreatic carcinoma

## Abstract

Ellagic aicd (EA), a dietary polyphenolic compound found in plants and fruits, possesses various pharmacological activities. This study investigated the effect of EA on human pancreatic carcinoma PANC-1 cells both in vitro and in vivo; and defined the associated molecular mechanisms. In vitro, the cell growth and repairing ability were assessed by CCK-8 assay and wound healing assay. The cell migration and invasion activity was evaluated by Tanswell assay. In vivo, PANC-1 cell tumor-bearing mice were treated with different concentrations of EA. We found that EA significantly inhibited cell growth, cell repairing activity, and cell migration and invasion in a dose-dependent manner. Treatment of PANC-1 xenografted mice with EA resulted in significant inhibition in tumor growth and prolong mice survival rate. Furthermore, flow cytometric analysis showed that EA increased the percentage of cells in the G1 phase of cell cycle. Western blot analysis revealed that EA inhibited the expression of COX-2 and NF-κB. In addition, EA reversed epithelial to mesenchymal transition by up-regulating E-cadherin and down-regulating Vimentin. In summary, the present study demonstrated that EA inhibited cell growth, cell repairing activity, cell migration and invasion in a dose-dependent manner. EA also effectively inhibit human pancreatic cancer growth in mice. The anti-tumor effect of EA might be related to cell cycle arrest, down-regulating the expression of COX-2 and NF-κB, reversing epithelial to mesenchymal transition by up-regulating E-cadherin and down-regulating Vimentin. Our findings suggest that the use of EA would be beneficial for the management of pancreatic cancer.

## INTRODUCTION

Pancreatic cancer ranks the fourth most deadly form of cancer in the United States and it has a poor prognosis with a 5-year survival rate of less than 5% [1]. The median survival time of pancreatic cancer is less than 6 months and the overall survival time is less than 2 years due to its high malignant degree, aggressive local invasion and early distal metastasis [2]. Because pancreatic cancer has a poor response to conventional chemotherapy and radiotherapy, there is a growing interest in natural compounds for enhancing pancreatic cancer prevention and treatment. Recent studies have highlighted the significance of dietary factors in managing epithelial malignancies including pancreatic cancer [3].

Ellagic acid (EA), a natural dietary polyphenolic compound, is present in several plants and fruits, including pomegranates, strawberries, raspberries and blackberries [4]. It has antioxidant, antifibrosis, and anticarcinogenic properties [5–7]. The anticarcinogenic effect of EA was shown in several types of cancers including colon, breast, and prostate cancers [8]. In spite of these findings, the effects of EA on pancreatic cancer have not been studied. Furthermore, the mechanisms mediating anticancer effect of EA remain unknown.

NF-κB and COX-2 are two main factors in inflammatory reaction, and are associated with tumorigenesis and tumor progression [9]. Epithelial-messenchymal transition (EMT) is a basic step among various factors in tumor metastasis and invasion [10]. E-cadherin, as a prominent protein in epithelial cells is down-regulated, and Vimentin, a prominent mesenchymal protein in interstitial cells, is up-regulated during EMT [11].

The purpose of this study was to investigate the effect of EA on the human pancreatic carcinoma PANC-1 cells and pancreatic cancer in Balb c nude mice and to define the associated molecular mechanisms. PANC-1 cells were chosen because they are one of the drug-resistant pancreatic cancer cells and are much more resistant to gemcitabine relative to other pancreatic cancer cells. They have strong survival ability and can survive for over 3 days even in the complete absence of nutrients such as glucose, amino acids, and serum. Our data showed that EA significantly inhibited PANC-1 cell growth, repairing activity, migration and invasion. EA also inhibited the growth of tumor xenograft in Balb C nude mice. The molecular mechanisms under the cell growth inhibitory effect of EA might be related to the suppression of NF-κB and COX-2; and the reverse of EMT in pancreatic cells. Our results imply that EA may offer therapeutic benefits against pancreatic cancer.

## RESULTS

### EA inhibits pancreatic cancer cell growth

**Figure 1:**
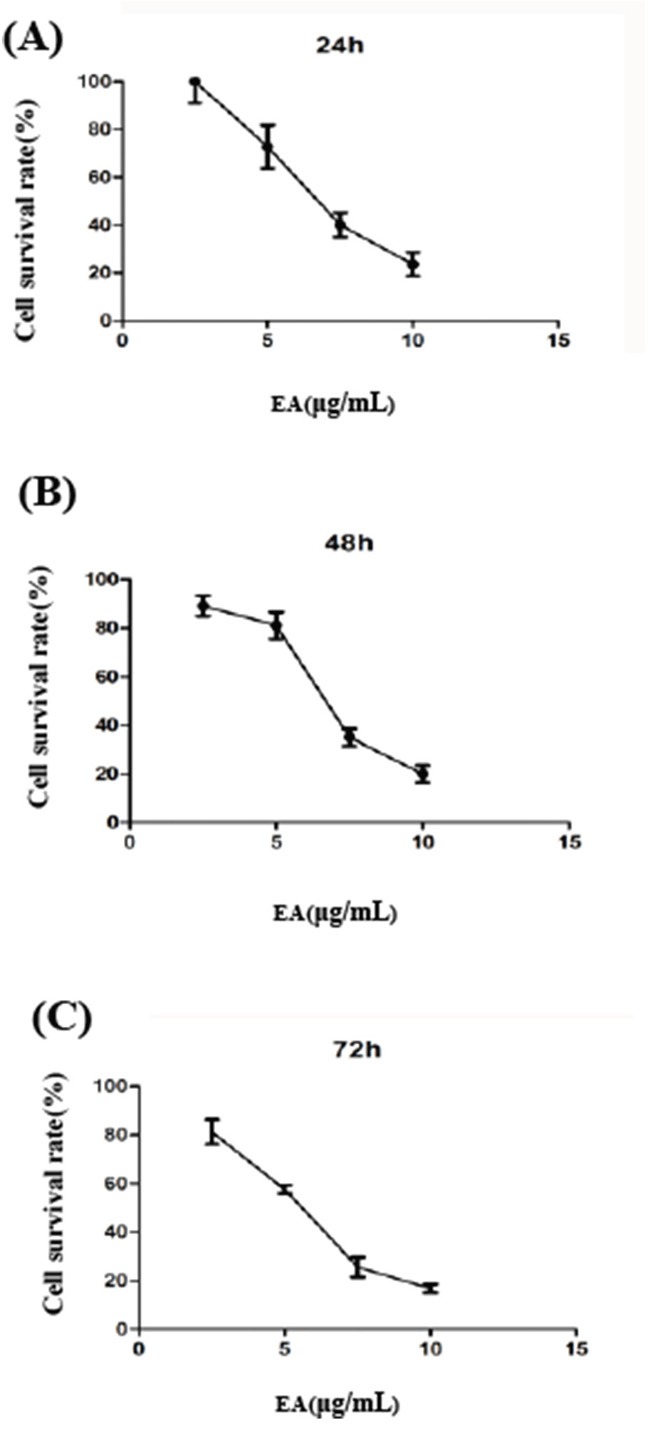
Ellagic acid inhibits the growth of PANC-1 cells Cells were incubated with various concentration of ellagic acid (0, 2.5 μg/ml, 5.0 μg/ml, 7.5 μg/ml and 10.0 μg/ml) and cultured for 24 h **A**., 48h **B**. and 72 h **C**. CCK-8 assay was performed. EA=Ellagic acid.

To evaluate the effects of EA on the growth of PANC-1 cells, cells were incubated with various concentration of EA (0,2.5μg/ml, 5.0μg/ml, 7.5μg/ml and 10.0μg/ml) for 24h (A), 48h (B) and 72h (C). CCK-8 assay was performed and the result showed that the cell survival rate decreased with the increase of EA concentration. We observed that the half maximal inhibitory concentrations (IC50) of EA were 7.38μg/ml, 6.42μg/ml and 5.41μg/ml for 24h, 48h and 72h respectively. This result indicated that EA inhibited pancreatic cancer cell growth in a dose-dependent manner (Figure 1A, 1B and 1C).

### EA inhibits the repairing ability of PANC-1 cells

**Figure 2:**
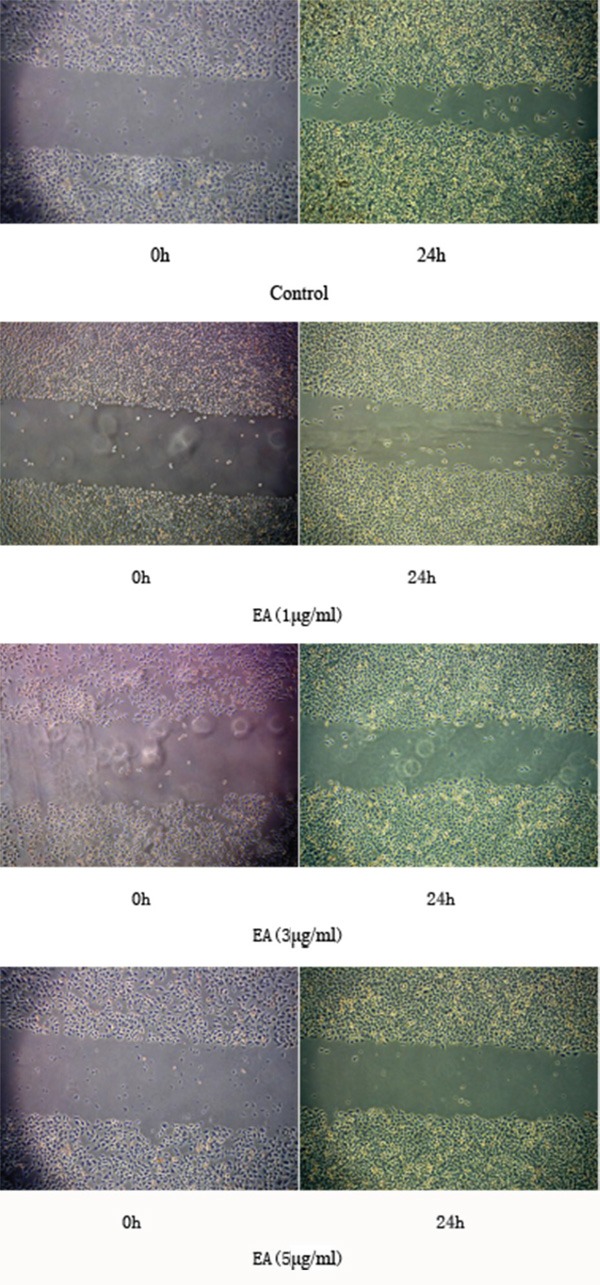
EA inhibits the healing ability of the PANC-1 cells Cells were cultured for 24h, incubated with various concentration of ellagic acid (0, 1μg/ml,3 μg/ml,5 μg/ml). Wound healing was observed under the microscope(×50).

Based on the above result, the IC50 of EA is greater than 5 μg/ml. Therefore EA was used in the concentration under 5 μg/ml in the following experiments. In order to evaluate the effect of EA on the repairing ability of PANC-1 cells, wound healing assay was performed. A scratch wound was created on monolayer PANC-1 cell culture and the cultures were treated with 0, 1, 3 and 5μg/ml of EA. The result showed that wound healing occurred in the control group while those in the EA treated groups, the wound healing decreased with the increased concentration of EA (Figure 2). This result suggested that EA inhibited the repairing ability of PANC-1 cells with concentrations lower than its IC50.

### EA inhibits the migration and invasion of PANC-1 cells

**Figure 3:**
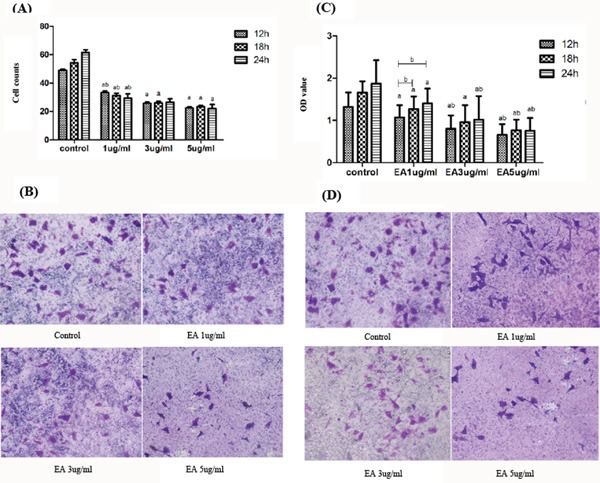
Effect of EA on cell migration and invasion **A**. After treated with different concentration of ellagic acid for 12, 18 or 24 h, the number of cells penetrating the membrane on the bottom of the upper compartment. **B**. the morphology of cells penetrating the membrane on the bottom of the upper compartment observed under the microscope (×200). **C**. OD value of the cell suspension washed from the bottom of the upper compartment. **D**. After treated with different concentration of ellagic acid for 24 h, the cells penetrating the matrigel on the bottom of the upper compartment observed under the microscope(×200). Data represent the mean ± SD. ^a^P<0.05 between different groups on the same time, ^b^P<0.05 between different groups on the same concentration.

The effect of EA on cell migration and invasion of PANC-1 cells was evaluated using Transwell chambers. We counted the cells penetrating the membrane on the bottom of the upper compartment to assess the cell migration (Figure 3A and 3B). To examine the cell invasion ability, cells were cultured in Transwell chambers with Matrigel. The cells that penetrated through the Matrigel in the bottom of the upper compartment were counted. Cells were also collected and the light absorbance of cell suspension was read at 490 nm in a plate reader (Thermo Scientific, Waltham, MA, USA). The inhibition rate (%) of cell invasion was calculated as follows (Figure 3C and 3D):

Inhibition rate (%) = (OD490_control_-OD490_sample_)/OD490_control_×100%

### EA inhibits the growth of PANC-1 cell xenografts in Balb c nude mice

**Figure 4:**
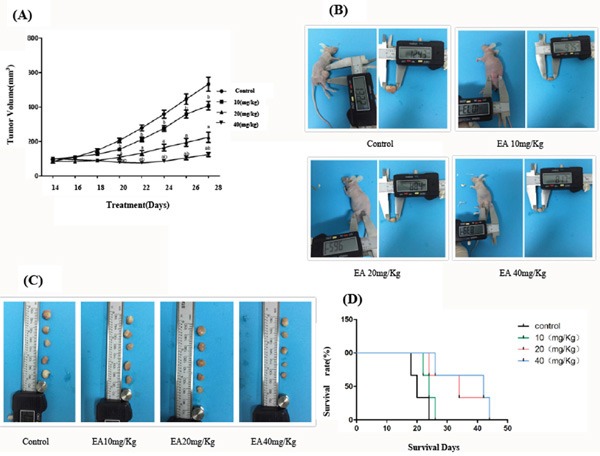
EA inhibits the growth of PANC-1 xenografts in Balb c nude mice **A**. Tumor bearing mice were treated with EA(0, 10 mg/kg, 20 mg/kg and 40 mg/kg body weight) through gavage. Tumor volume of mice was recorded daily. Data represent the mean ± SD. ^a^P<0.05 between different groups on the same time, ^b^P<0.05 between different groups on the same concentration. **B**. Size of tumors. Photographs of tumors of mice were taken at the end of experiment. **C**. Size of tumors. Photographs of tumors derived from control and EA-treated mice were taken at the end of experiment. **D**. Tumor bearing mice were treated with EA(0, 10 mg/*kg*, 20 mg/*kg* and 40 mg/*kg* body weight) through gavage once every two days until death. The survival rate and survival days of mice were recorded.

To examine the in vivo effect of EA on the growth of tumor, the effects of EA on the growth of PANC-1 cell xenografts in nude mice was investigated. The result showed that EA inhibited the PANC-1 tumor growth (volume and size) in Balb c nude mice (Figure 4A, 4B and 4C). It was also found that the mice survival rate increased with the increase of the concentration of EA (Figure 4D). We did not found any toxicity of EA in the liver, spleen and intestine of mice treated with EA, suggesting that EA at the concentration tested is safe in animals.

### EA increases the percentage of cells in the G1 phase of the cell cycle

**Figure 5:**
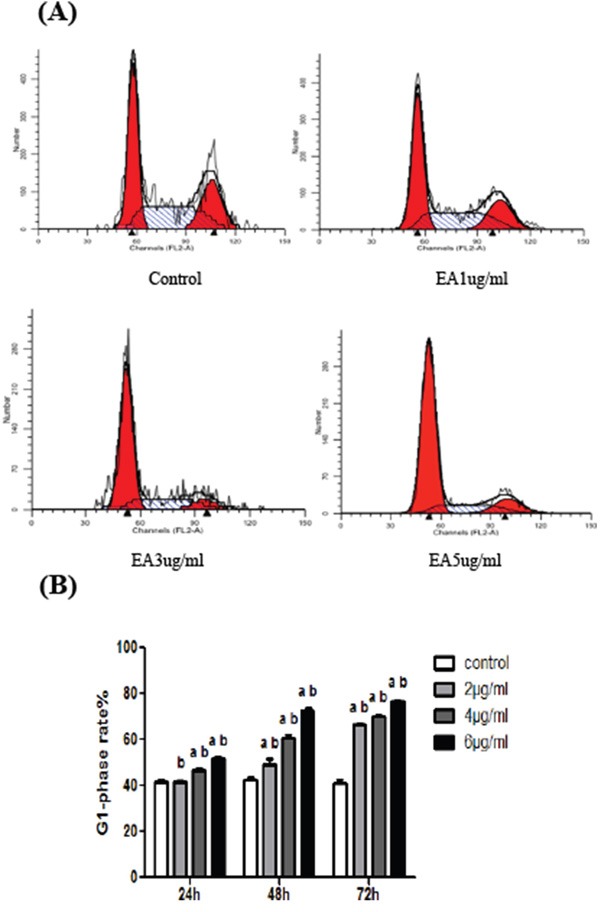
EA increases the percentage of cells in the G1 phase of cell cycle **A**. After treated with different concentration of ellagic acid for 48 h, the cellular DNA content of cells was analyzed by flow cytometry. **B**. After treated with different concentration of ellagic acid for 24, 48 or 72 h, we analyzed the cellular DNA content of cells by flow cytometry and the percentage of cells in the G1 phase was calculated. Data represent the mean ± SD. ^a^P<0.05 between different groups on the same time, ^b^P<0.05 between different groups on the same concentration.

The above results indicated that EA inhibits pancreatic cell growth both in vitro and in vivo. To evaluate the mechanism underlying the cell inhibitory effect of EA, the effect of EA on the cell cycle was investigated. We examined the cell cycle phase distribution of cells treated with EA using flow cytometry after PANC-1 cells were stained with PI and RNase. The result showed that EA significantly increased the percentage of cells in the G1 phase of the cell cycle. This result suggested that EA inhibits the cell proliferation by inducing cell cycle arrest (Figure 5A and 5B).

### EA down-regulates the expression of COX-2, NF-κB, Vimentin and up-regulates the expression of E-cadherin

**Figure 6:**
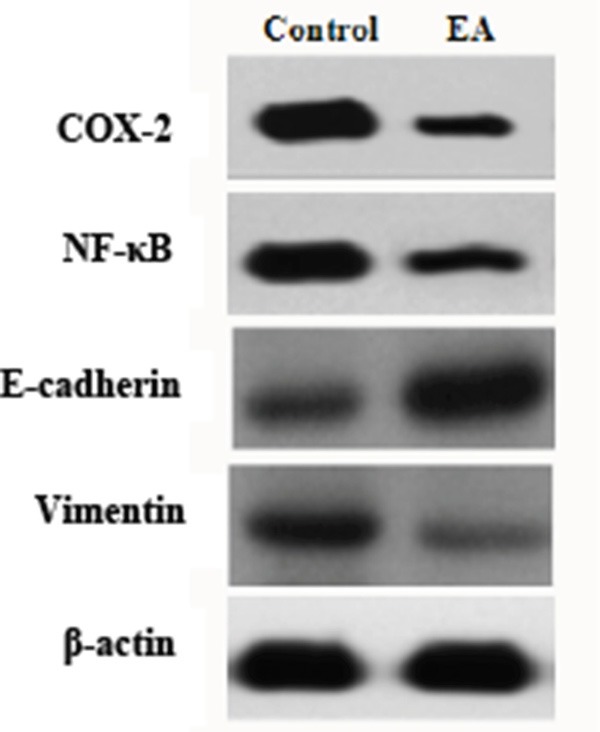
Western blot analysis on the expression of COX-2, NF-κB, E-cadherin and Vimentin The β-actin was used as a loading control. A representative picture was shown from 3 independent experiments.

To further explore the mechanisms underlying the cell inhibitory effect of EA, the expression of a number of molecules that regulated tumor development and progression was investigated by Western blot analysis. We found that EA treatment down-regulated the expression of COX-2, NF-κB, Vimentin and induced the expression of E-cadherin in PANC-1 cells (Figure 6). Because COX-2 and NF-κB are the two main activating factors in inflammatory reaction associated with tumor development and progression. E-cadherin and Vimentin are proteins in epithelial mesenchymal transition (EMT) which is closely related with tumor invasion and metastasis. These results suggested that the cell growth inhibitory effect of EA might be regulated by COX-2 and NF-κB pathways, and treatment with EA might prevent EMT therefore inhibit tumor invasion and metastasis.

## DISCUSSION

Pancreatic ductal adenocarcinoma (PDA) remains a lethal human malignancy with historically limited success in treatment [12–14]. Because pancreatic cancer is hypovascular with a dense fibrous capsule on the surface, chemotherapeutic drugs are difficult to permeate to the tumor. Also because of its natural or acquired drug resistance, pancreatic cancer is not always sensitive to chemotherapeutic drugs in clinical practice [15]. Natural products attract a lot of attention in recent years because of fewer side effects and definite therapeutic efficacy in the treatment of tumors [16].

The present study investigated the effect of the natural drug EA on human pancreatic carcinoma PANC-1 cells and pancreatic xenograft tumor in Balb c nude mice. In CCK-8 assay, we found that EA inhibited cell growth in a dose-dependent manner. In wound healing assay, EA inhibited the repairing ability of the PANC-1 cells. We also found that EA inhibited the migration and invasion of PANC-1 cells through Transwell chamber. In mouse tumor xenografts, EA inhibited the PANC-1 tumor growth (volume and size) in Balb c nude mice and the mice survival rate increased with the increased concentration of EA treatment. In addition, we found that EA has no notable toxicity in the liver, spleen and intestine in mice treated with EA, indicating that EA is a safe natural drug.

G1/S and G2/M are the two most important checkpoints in cell cycle and the key regulation lies in whether the cells block or through the checkpoints [17]. G1 phase arrest is the important step in cell proliferation inhibition and if cells can not get into the S phase, DNA will not be synthesized and the protein metabolism will be disturbed, then the division of cancer cells will be inhibited [18]. We observed the effect of EA on the cell cycle by flow cytometry and our data showed that EA significantly increased the percentage of cells in the G1 phase of the cell cycle. Therefore cell cycle arrest might be one of the mechanisms that EA inhibits the proliferation of PANC-1 cells.

COX-2 is the key enzyme that catalyzes arachidonic acid into prostaglandin [19] and has an important biological function in the occurrence and development of many types of tumors. Recent studies indicate that as one of the important inflammatory factors, COX-2 plays an important role in the development of chronic pancreatitis and pancreatic cancer. It is generally accepted that under a series of carcinogenic factors, normal pancreatic ductal epithelial cells first turn into pancreatic intraephithelial neoplasm and then develop into aggressive pancreatic ductal adenocarcinoma, and the inflammatory response plays an important role in promoting the evolution [20–21]. The occurrence and development of pancreatic cancer is closely related to the high expression of COX-2 [22–23], the related mechanisms include inducing proliferation, inhibiting apoptosis and inducing the expression of VEGF et al [24].

NF-κB, as another important inflammatory factor, can activate the growth and differentiation of epithelial cells through regulating the expressions and activities of various cytokines and chemokines related to inflammation. NF-κB inhibits the apoptosis of pancreatic cells through regulating a variety of apoptosis related genes, including Bcl-2, Bcl-xL and caspase-3 [25–26]. A number of chemotherapeutic drugs, including gemcitabine, can activate NF-κB and it may be the reason that pancreatic cancer is not sensitive to gemcitabine [27]. Therefore, targeting NF-κB and inhibiting its activity is becoming a target of research for the prevention and treatment of pancreatic cancer. Our results that EA down-regulates the expression of COX-2 and NF-κB in pancreatic cancer cells will provide a rational for EA to become a new chemotherapeutic agent against pancreatic cancer.

EMT has a great clinical significance in the invasion and metastasis of epithelial tumors. The decreased expression of E-cadherin and the increased expression of Vimentin are the indications of EMT. Low expression of E-cadherin is significantly related to poor tumor differentiation, invasion and metastasis [28], and this might be regulated by activating its transcription factors, such as Snail, SIP1 and Slug [29]. Recent studies showed that knockout Vimentin in malignant cells decreased the ability of tumor cell invasion and metastasis [30]. The increased expression of E-cadherin and the decreased expression of Vimentin in this study indicated that EA might have the anti-EMT effect.

In summary, our study provides important information regarding the antitumor activities of EA in human pancreatic cancer cells. Specifically, we have demonstrated that EA inhibits cell growth, cell repairing activity, migration and invasion of PANC-1 cells. EA also inhibited the PANC-1 tumor growth (volume and size) in Balb c nude mice and the mice survival rate was increased with the increased concentration of EA treatment. The mechanisms underlying the anti-tumor effect of EA might be due to EA can induce cell cycle arrest, inhibit the expression of COX-2, NF-κB, Vimentin and induce the expression of E-cadherin. Therefore, EA can be a promising natural product for the treatment and prevention of pancreatic cancer.

## MATERIALS AND METHODS

### Chemicals and reagents

Ellagic acid, Propidium iodide (PI), RNase A, Cell counting kit-8(CCK-8) and Transwell chamber were purchased from Sigma Chemical (St. Louis, MO). Fetal bovine serum (FBS) was from GIBCO (Carlsbad, CA). Antibodies against COX-2, NF-κB, E-cadherin, Vimentin and β-actin were purchased from Cell Signaling Technology (Danvers, MA, USA). Enhanced chemiluminescence (ECL), Western blot detection reagents were purchased from American Life Sciences Inc. (Arlington Heights, IL).

### Cell culture

PANC-1 cells were purchased form American Type Culture Collection (ATCC) (Manassas, VA). These cells were maintained in Dulbecco's Modified Eagle's medium (DMEM) supplemented with 10% heat-inactivated FBS, 100 U/mL of penicillin and 100 μg/mL of streptomycin (all from Gibco BRL, San Diego, CA, USA) at 37°C in a 5 % CO2 atmosphere.

### Cell growth assessment

CCK-8 assay was used to assess the inhibitory effect of EA on PANC-1 cells according to manufacture's instruction. In brief, PANC-1 cells (1 × 10^4^ in 100 μL) were seeded on 96-well plates. After overnight growth, cells were incubated with various concentration of EA at 37°C for 24, 48 or 72 h, respectively. Then 10 μL of CCK-8 was added to each well, incubated at 37°C for an additional 3h and then read immediately at 490 nm by a plate reader (Thermo Scientific, Waltham, MA, USA). The inhibition rate (%) of cell activity was calculated as follows:

Inhibition rate (%) = (A490_control_-A490_sample_)/A490_control_×100%

The half maximal inhibitory concentrations (IC 50) were calculated using GraphPad Prism 5.

### Cell repairing ability assessment

PANC-1 cell repairing ability was evaluated using wound healing assay. Cells (1 × 10^4^ in 100 μL) were seeded on 6-well plates for 24h. A scratch was drawn in these cells with a blunt head and cells were washed three times. At last, these cells were incubated with various concentration of EA at 37°C for 24 and the cell repairing ability was observed.

### Cell migration and invasion assay

The ability of migration and invasion of PANC-1 cells was examined using Transwell chambers. For cell migration assay, cells (1 × 10^4^ in 100 μL) were seeded on the upper compartment of 12-well Transwell chambers and incubated with various concentration of EA for 24h. The under compartment contained DMEM supplemented with 10% heat-inactivated FBS. After 24h, the medium on the upper compartment was removed carefully and the cells migrated through the membrane were fixed with 95% ethanol for 10 minutes. Then the upper compartment was token out and put in a solution with 1% crystal violet at 4°C for 6h. After staining, the upper compartment was washed and the cells migrated through the membrane on the bottom of the upper compartment were counted under the inverted microscope. For cell invasion assay, matrigel was put between the upper and under compartments. The above procedures were performed and cells migrated through the matrigel was evaluated.

### Anti-tumor effect of EA on the pancreatic cancer cell xenograft in Balb C nude mice

Balb C nude mice (4-6 weeks old) were purchased from the National Cancer Institute (Frederick, MD). Mice were raised in specific pathogen free (SPF) environment with clean laminar flow frame. Room temperature was adjusted to (25±1)°C, relative humidity 40% to 60% in Nanjing Drum Tower Hospital (Nanjing Drum Tower Hospital, The Affiliated Hospital of Nanjing University Medical School) animal experiment center (Use license: SCXK (SU)2014—0052).) To establish a mouse pancreatic cancer xenograft model, PANC-1 cells (2× 10^6^ cells) mixed with Matrigel (Becton Dickinson, Bedford, MA) at 50:50 ratio in a final volume of 75μL were injected subcutaneously into the flanks of Balb C nude mice as per approved protocol. Mice were divided into 4 groups, 5 mice per group. In order to examine the anti-tumor potential of EA, mice were treated with EA (0, 10 mg/kg, 20 mg/kg and 40 mg/kg body weight) through gavage for 2 weeks after tumor formation. At the end of the experiment, mice were euthanized and tumors were isolated and weighted.

### Flow cytometric analysis of the cell cycle

Cell cycle analysis was conducted by using a Cell Cycle Analysis Kit (Beyotime, Shanghai, P.R. China) following the manufacturer's instruction. Briefly, after treatment with and without EA for 24, 48 or 72 h, PANC-1 cells were harvested. PANC-1 cells were trypsinized and centrifuged at 1,500×*g* for 5 min. The supernatant was removed and the cells were washed with phosphate buffered saline (PBS) and fixed with 70% ethanol at 4°C for 24 h. The cells were then washed and stained with a solution containing 50 μg/mL of PI and 100 μg/mL of RNase A at 37°C for 30 min in the dark. The cellular DNA content and cell cycle phase distribution were analyzed using flow cytometry (Beckman Coulter, Epics XL).

### Western blot analysis

PANC-1 cells (5 × 10^6^) were treated with various concentrations of EA and vehicle respectively for 24, 48 or 72 h. Total protein extract from PANC-1 cells was prepared using cell lysis buffer. The lysates (30μg) was resolved on SDS-PAGE and electroblotted onto polyvinylidene difluoride membrane (PVDF, Millipore Corp., Bedford, MA) and immunblotted using various primary antibodies including COX-2 (1 : 1000), NF- κB (p65) (1 : 1000), E-cadherin (1 : 1000), Vimentin (1 : 1000) and β-actin (1 : 1000), and then incubated with corresponding horseradish-peroxidase-conjugated secondary antibodies. Western blot bands were visualized by incubation with ECL reagent (ThermoScientific Pierce, Waltham, USA) and exposure to Clinx ChemiScope system (Shanghai, P.R. China).

### Statistical analysis

The SPSS 17.0 statistical software (SPSS Inc., Chicago, IL) was applied for statistical analysis. All values were expressed as the mean ± SD and analyzed by one-way analysis of variance (ANOVA) followed by Tukey's Multiple Comparison Test. A P-value of less than 0.05 was considered statistically significant.
